# Cullin3 aggravates the inflammatory response of periodontal ligament stem cells via regulation of SHH signaling and Nrf2

**DOI:** 10.1080/21655979.2021.1943603

**Published:** 2021-06-30

**Authors:** Wanhong Chen, Jiangling Su, Shixiong Cai, Chun Shi

**Affiliations:** a Department of Stomatology, Quanzhou First Hospital, Quanzhou, Fujian, China; b Department of Endodontics and Periodontics, School of Stomatology, Dalian Medical University, Dalian, Liaoning, China

**Keywords:** *P. gingivalis*-LPS, CUL3, SHH, inflammation, PDLSCs

## Abstract

It is found that the activation of Sonic Hedgehog (SHH) signaling pathway is related to the degree of inflammation in patients suffering from periodontitis. Cullin3 (CUL3), an important ubiquitin ligase, can control SHH signaling. In this study, we were dedicated to clarify the roles of SHH and CUL3 in *P. gingivalis*-LPS (Pg-LPS)-treated periodontal ligament stem cells (PDLSCs). In this study, cell viability was detected using cell counting kit-8 (CCK-8). The inflammatory cytokines of PDLSCs were estimated by enzyme-linked immunosorbent assay (ELISA). With the application of western blots, the protein levels of SHH, Gli1 and NF-E2-related factor 2 (Nrf2) were determined. Alkaline phosphatase staining and Alizarin red staining were performed to evaluate the differentiation and mineralization capabilities of PDLSCs. The apoptotic cells were screened using TUNEL staining. The results showed that Pg-LPS inhibited cell viability and triggered inflammation of PDLSCs. Overexpression of CUL3 weakened the differentiation and mineralization capabilities of PDLSCs. Moreover, CUL3 overexpression aggravated inflammation and cell apoptosis induced by Pg-LPS. It is worth noting that although the protein levels of SHH, Gli1 and Nrf2 were elevated in PDLSCs treated with Pg-LPS, overexpression of CUL3 decreased the expressions of Gli1 and Nrf2. Overall, SHH/Gli1 and Nrf2 were involved in the inflammation and cell apoptosis of PDLSCs, which was dominated by CUL3.

## Graphical abstract

**Figure:**
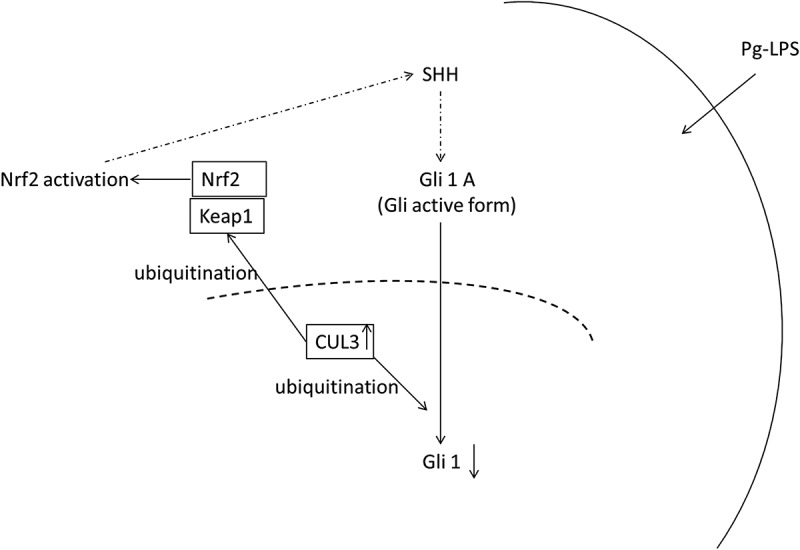

## Introduction

Periodontitis, a prevalent oral disease, has wide footprint in every corner of the world. Epidemiological surveys show that over 50% of adults suffer from periodontal disease [1,2]. It is generally accepted that excessive accumulation of plaque bacteria on the tooth surface invades periodontal tissue and triggers the inflammatory response, further resulting in the destruction of the periodontal tissue connection and the resorption of alveolar bone [3]. What’s worse, the supporting structure of the tooth disappears [4]. However, periodontal ligament has good regeneration ability in physiological conditions so that alveolar bone and periodontal ligament are always in dynamic reconstruction, thus maintaining the integrity of periodontal support structure [5].

At present, the effective therapies for periodontitis are in scarcity [6]. Nowadays, the biological role of periodontal ligament stem cells (PDLSCs) has been a hotspot in the treatment of periodontal disease. PDLSCs act as an important player in maintaining the dynamic balance of periodontal tissues, at the same time, they also serve as the basis of periodontal tissue regeneration due to their multiple differentiation capabilities [7]. Nevertheless, osteogenic differentiation of PDLSCs was inhibited during the progression of periodontitis [8].

Sonic Hedgehog (SHH), a member of Hedgehog (Hh) family, is widely distributed in multiple organs [9]. SHH signals regulate the formation of a variety of tooth components, including enamel, dentin, cementum and other soft tissue. In addition, dental mesenchymal cells positive for Gli1, a downstream transcription factor of Shh signaling, have been found to have stem cell properties, including multipotency and the ability to self-renew [10]. Hh signal is usually silent in adults, even though it is involved in tissue maintenance and regeneration by modulating the regeneration and differentiation of stem cells [11]. High expression of SHH was observed in gingival crevicular fluid of patients with periodontitis [12], indicating that SHH may participate in the inflammatory response of periodontitis. Multiple studies have shown that the SHH signaling pathway can inhibit the production of inflammation in disease and inhibit cell apoptosis, moreover, SHH signaling pathway plays an important role in cell differentiation and regeneration [13–15].

E3 ubiquitin ligase is the largest ubiquitin ligase family, which can be divided into HECT domain, RING domain and U-box domain according to their different structures. As a scaffold protein with RING domain, CUL3 is involved in the formation of the ubiquitin ligase complex, which binds to the adaptor protein with the BTB domain and has a unique 3-box domain to stabilize the binding site, thus specifically recognizing the substrate and completing the ubiquitination modification of the substrate protein [16]. CUL3 differs from other CUL in that it binds directly to the substrate recognition subunit BTB domain without the need for additional bridging protein connections [17]. Therefore, there are not many active CUL3 ubiquitin ligase complexes [18,19].

CUL3 was the representative of a common signaling node for controlling SHH signaling pathways [20]. SHH is most widely distributed in human tissue cells and is involved in genes transcription, regulation of cytokine and functional protein expression [21]. The SHH signaling pathway is composed of SHH protein, Gli1 protein and so on. Studies showed that as a key transcription factor responsible for SHH signal, Gli1 could be degraded by CUL3 [22,23]. In addition, Keleh-like ECH-associated protein 1 (Keap1) could interact with CUL3 as well as mediate the ubiquitination and degradation of NF-E2-related factor 2 (Nrf2) [24,25]. Keap1/Nrf2 serves as a crucial signaling pathway that orchestrates inflammation and oxidative stress [26,27].

From the above, we speculated that CUL3 plays an important role in periodontitis model cells and plays a regulatory role through the SHH/Gli signaling pathway and Nrf2. In this study, we aimed to illustrate the roles of SHH signaling pathways in LPS-induced PDLSCs and evaluate the effect of CUL3 in the inflammatory process. And we want to provide a theoretical basis for targeted treatment of periodontitis.

## Methods and materials

### Cell culture and transfection

PDLSCs (LMAI Bio, Shanghai, China) were maintained in osteogenic-inducing medium containing 10% FBS, 50 μM/ml ascorbic acid, 5 mM β-glycerophosphate and 100 nM dexamethasone in a humidified incubator at 37°C with 5% CO_2_. *P. gingivalis*-LPS (Pg-LPS; Sigma-Aldrich, MA, USA) was used to stimulate PDLSCs as previously described [28,29]. Overexpression plasmids pcDNA 3.1-CUL3-1 (pcDNA-CUL3-1 group), pcDNA 3.1-CUL3-2 (pcDNA-CUL3-2 group) and empty plasmid (pcDNA-NC group) were generated by GenePharma (Shanghai, China). About 1.2 μg pcDNA 3.1-CUL3-1, pcDNA 3.1-CUL3-2 and empty plasmid were transfected respectively into PDLSCs at a density of 2 × 10^4^ per well using Lipofectamine 3000 reagent (Invitrogen) according to the instruction [30]. RT-qPCR was used to determine the cell transfection efficiency. Control group does nothing. We then divided the cells into control, LPS, LPS+pcDNA-NC and LPS+pcDNA-CUL3-1. LPS (1 μg/mL) was exposed to cells transfected pcDNA-NC or pcDNA-CUL3-1.

### CCK-8 assay

PDLSCs were seeded into a 96-well plate at a density of 1 × 10^3^ per well. At first, cell viability was estimated at 0 h, 24 h and 48 h, then 10 μl CCK-8 solution (Dojindo, Kumamoto, Japan) was added into each well, followed by incubation for 1 h. The absorbance value was at last recorded at 450 nm [31].

### ELISA assay

The supernatants of PDLSCs were collected and centrifuged at 4°C (1000 g) for 10 min. The concentration of TNF-α, IL-6, IL-1β and IL-10 was determined by ELISA kits (Beyotime, Jiangsu, China) according to the manufacturer’s instruction. The absorbance value was recorded at 450 nm.

### Western blots

Western blot was carried out according to the protocols previously reported [32]. Total protein from cells were extracted using RIPA lysis buffer (Solarbio, Beijing, China). The protein concentration was determined with the application of a BCA assay kit (Beyotime, Jiangsu, China). Afterward, SDS-PAGE was prepared to separate proteins, and then the latter were transferred onto polyvinylidene difluoride (PVDF) membranes (EMD Millipore, MA, USA). PVDF membranes were incubated with primary antibodies, including anti-SHH (1:1000, ab53281, Abcam, Cambridge, UK), anti-Gli1 (1:1000, ab134906, Abcam, Cambridge, UK), anti-CUL3 (1:1000, ab75851, Abcam, Cambridge, UK), anti-Nrf2 (1:1000, ab62352, Abcam, Cambridge, UK), anti-NQO1 (1:1000, ab80588, Abcam, Cambridge, UK) and Anti-GAPDH (1:1000, ab8245, Abcam, Cambridge, UK), and were removed the second day. In addition, secondary antibodies (Sigma-Aldrich) were prepared for incubation with PVDF membranes. The protein bands were visualized and analyzed using a chemiluminescence system (Bio-Rad, CA, USA).

### Alkaline Phosphatase (ALP) staining

PDLSCs were seeded into a 24-well plate at a density of 2 × 10^4^ per well. Osteogenic-inducing medium was prepared to culture PDLSCs for 7 d. The medium was refreshed every 3 days and removed later on the 7th day. PDLSCS were subsequently cultured with added fix solution (MKBio, Shanghai, China) for 4 min at room temperature. Then with 0.6 ml dye (MKBio) added into each well, the cells were cultured at room temperature for 10 min in the dark. Cell observation was done under a light microscope (Carl Zeiss, Jena, Germany) at last. The absorbance of the cell cultures was measured at 405 nm and the final calcium level was normalized according to the total protein concentration in duplicate plates [33].

### Alizarin red staining

The mineralization of PDLSCs was evaluated by Alizarin red S. PDLSCs were briefly seeded into a 24-well plate at a density of 2 × 10^4^ per well. Osteogenic-inducing medium was utilized to culture PDLSCs for 21 d, after which PDLSCs were fixed by 4% paraformaldehyde for 15 min at 4°C. The cells were then incubated for 30 min with 0.2% Alizarin Red S solution (Sigma-Aldrich) added into each well. Lastly, cell observation was done under a light microscope (Carl Zeiss, Jena, Germany). The absorbance of the cell cultures was measured at 570 nm [34].

### TUNEL assay

TUNEL assay was carried out according to the protocols previously reported [35]. Apoptotic cells were detected by a TUNEL staining kit (KeyGEN, Jiangsu, China). Four percent paraformaldehyde was used to fix PDLSCs for 30 min at room temperature, after which Proteinase K was added for incubation for another 30 min at 37°C. Subsequently, treatment of 0.1% Triton X-100 was added for incubation for 5 min. Then the cells were equilibrated with 100 μL Equilibration buffer for 10 min at room temperature. Cells were labeled with 50 μL TdT reaction mix at 37°C for 1 h. after the stop buffer was added the nuclei were stained with DAPI. Finally, the cells were observed under a light microscope (Carl Zeiss, Jena, Germany).

### Statistical analysis

Data were presented as mean ± SD and analyzed by GraphPad Prism 6.0. Student’s t-test and one-way analysis of variance tests followed by Tukey’s post hoc test were used to compare the differences between groups. P < 0.05 was considered as statistical significance.

## Results

### Pg-LPS impairs cell viability and triggers inflammation of PDLSCs

**Figure 1.**
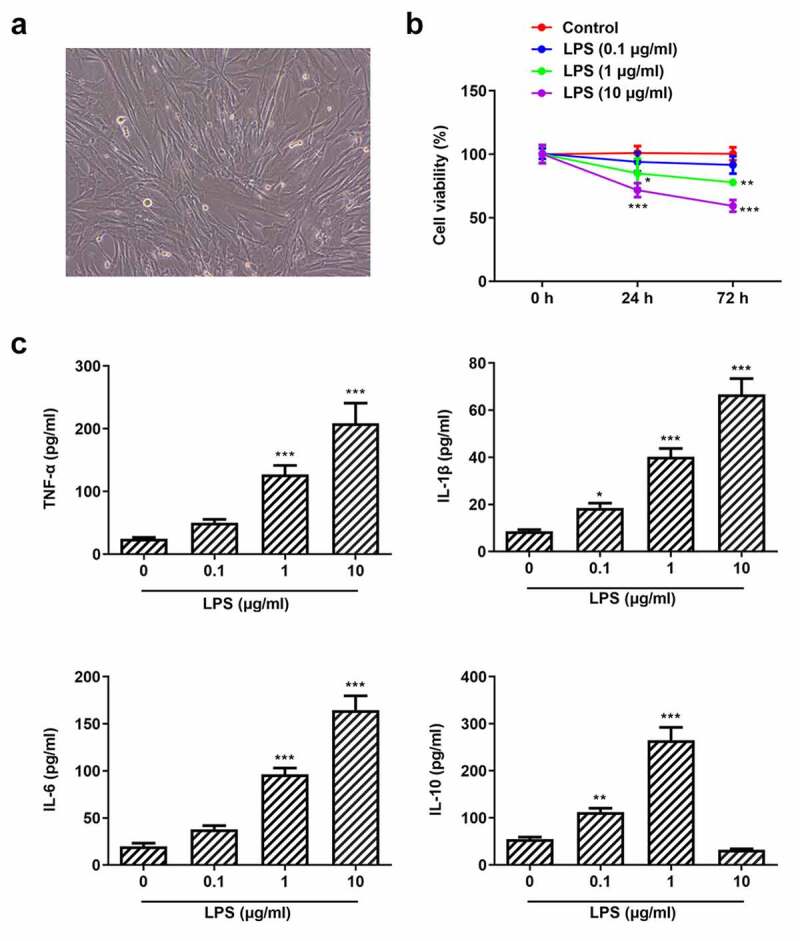
Pg-LPS impairs cell viability and triggers inflammation of PDLSCs. (a) Morphology of PDLSCs cells. (b) Effects of different concentrations of pg-LPS on the activity of PDLSCs were detected by CCK-8 assay. (c) Effects of different concentrations of pg-LPS on inflammatory factors (TNF-α, IL-6, IL-1β and IL-10) in PDLSCs were detected by ELISA assay. *p < 0.05, **p < 0.01 and ***p < 0.001 vs. 0 μg/ml

Studies have shown that Pg-LPS can induce the inflammatory response of human periodontal ligament stem cells and form a periodontal disease model [36,37]. In this study, the cell morphology of PDLSCs is shown in Figure 1(a) and PDLSCs were exposed to different concentrations of Pg-LPS, the viability of which was examined after 24 and 72 h. It was observed that cell viability was slightly decreased when cells were exposed to 0.1 μg/ml Pg-LPS. Of note, cell viability was significantly reduced when the concentration of Pg-LPS reached 1 μg/ml (Figure 1(b)). Pg-LPS at 10 μg/ml represented more severe damage on cell viability. In addition, the expressions of pro-inflammatory cytokines including TNF-α, IL-6 and IL-1β were elevated when PDLSCs were exposed to 1 μg/ml LPS, whereas the level of anti-inflammatory cytokines IL-10 was also increased following the exposure to 0.1 μg/ml or 1 μg/ml Pg-LPS (Figure 1(c)). However, it was noticed that IL-10 was markedly reduced when the concentration of Pg-LPS reached 10 μg/ml, indicating that high concentration of Pg-LPS predominantly triggered the secretions of pro-inflammatory cytokines and inhibited the secretions of anti-inflammatory cytokine.

### SHH signaling and Nrf2 are activated in Pg-LPS-treated PDLSCs

**Figure 2.**
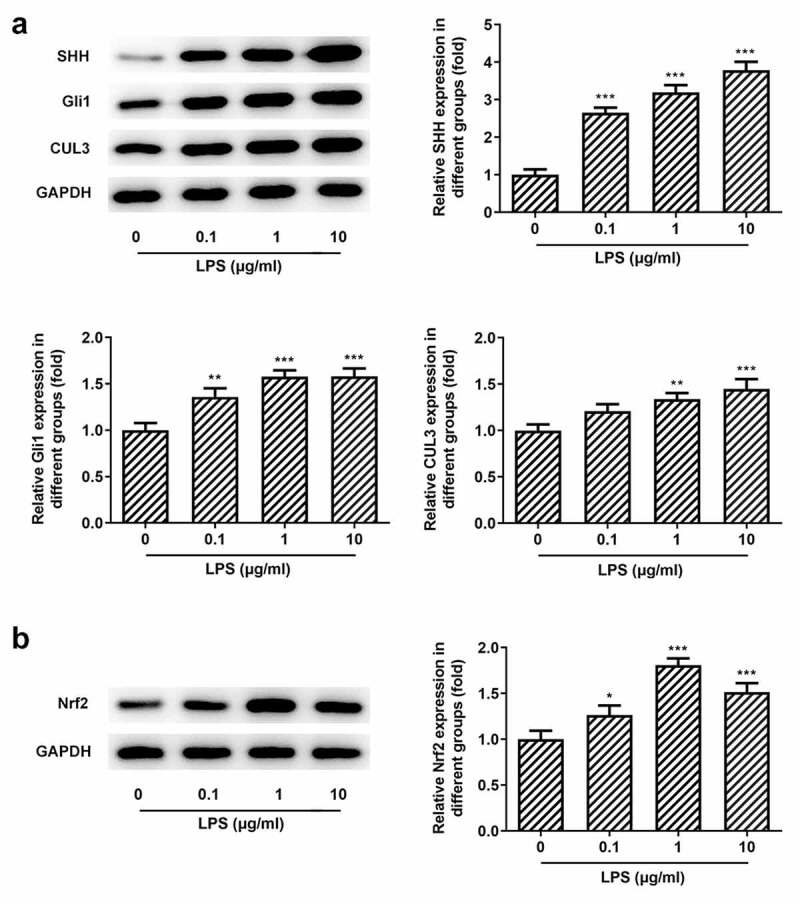
Effects of pg-LPS on SHH pathway-related proteins and Nrf2 in PDLSCs. (a) Western blot analysis was used to detect the effect of pg-LPS on SHH, Gli1 and CUL3 in PDLSCs. (b) Western blot analysis was used to detect the effect of pg-LPS on Nrf2 and NQO1 in PDLSCs. *p < 0.05, **p < 0.01 and ***p < 0.001 vs. 0 μg/ml. CUL3: Cullin3

We know that CUL3 plays a negative role in the regulation of the SHH signaling pathway through the degradation of activated Gli by ubiquitination [38]. Furthermore, CUL3 degrades Gli1, thereby affecting the regulation of Nrf2 on the SHH signaling pathway [39]. However, the regulatory role of CUL3,SHH/Gli, and NRF2 in periodontitis has not been reported so far. It was found that when the cells were exposed to Pg-LPS, the protein levels of SHH and Gli1 were elevated with the increase of Nrf2 and NQO1 (Figure 2(a,b)), which suggested that the stress of SHH, Gli1 and Nrf2 was increased in response to Pg-LPS challenge.

### Activity, differentiation and mineralization of PDLSCs are inhibited by CUL3 overexpression

**Figure 3.**
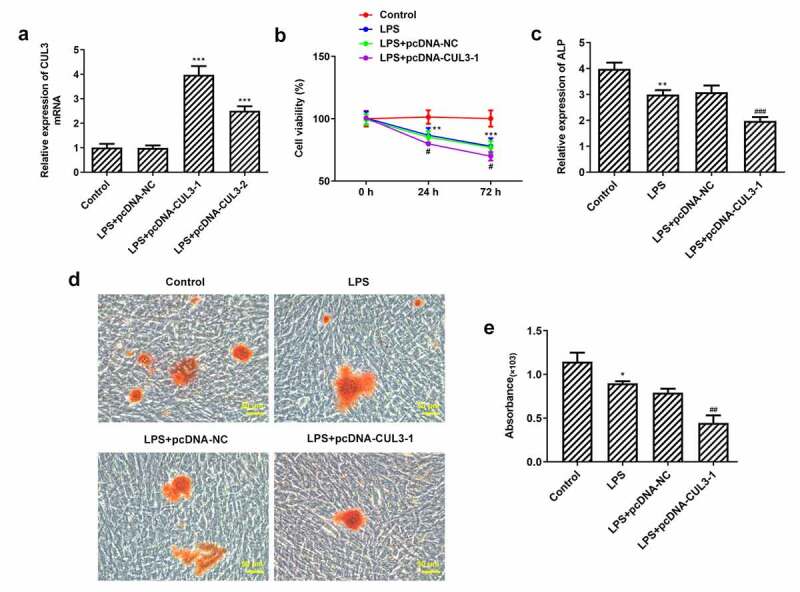
Activity, differentiation and mineralization of PDLSCs are inhibited by CUL3 overexpression. (a) pcDNA 3.1-CUL3-1, pcDNA 3.1-CUL3-2 and empty plasmid were transfected respectively into PDLSCs and the CUL3 overexpression efficiency was detected by qRT-PCR after transfection. ***p < 0.001 vs LPS+pcDNA-NC. (b) Effects of CUL3 overexpression on the activity of PDLSCs treated with 1 μg/mL pg-LPS were detected by CCK-8 assay. (c) Effect of CUL3 overexpression on cell differentiation of PDLSCs treated with 1 μg/mL pg-LPS was detected by ALP. (d) Effect of CUL3 overexpression on cell mineralization of PDLSCs treated with 1 μg/mL pg-LPS was detected by alizarin red staining. Magnification×200. (e) Statistical analysis of D. *p < 0.05, **p < 0.01,***p < 0.001 vs Control; #p < 0.05, ##p < 0.01, ###p < 0.001 vs LPS+pcDNA-NC. CUL3: Cullin3

Given that CUL3 plays a vital role in degrading Gli1 and Nrf2, overexpression plasmids pcDNA – CUL3-1 and pcDNA -CUL3-2 were transfected into PDLSCs. Results showed that compared with pc-DNA-NC group, the expression of CUL3 in pcDNA – CUL3-1 and pcDNA -CUL3-2 groups was increased and the trend was more obvious in PCDNA-CUL3-1 group (Figure 3(a)). So, we chose pcDNA-CUL3-1 for the following experiments. CCK-8 results showed that cell viability was significantly reduced in the LPS group compared with the control group. And overexpression of CUL3 further impaired cell viability with 1 μg/ml Pg-LPS (Figure 3(b)). The differentiation and mineralization capabilities of PDLSCs were estimated with the application of ALP and Alizarin red staining. The differentiation capability of PDLSCs induced by osteogenic-inducing medium was weakened when treated with 1 μg/ml Pg-LPS. Moreover, the overexpression of CUL3 further impaired the differentiation capability (Figure 3(c)). The result that CUL3 overexpression could impede PDLSCs mineralization was consistent with what we have observed via assessing the mineralization capability of PDLSCs (Figure 3(d,e)).

### Overexpression of CUL3 exacerbates inflammation and apoptosis

**Figure 4.**
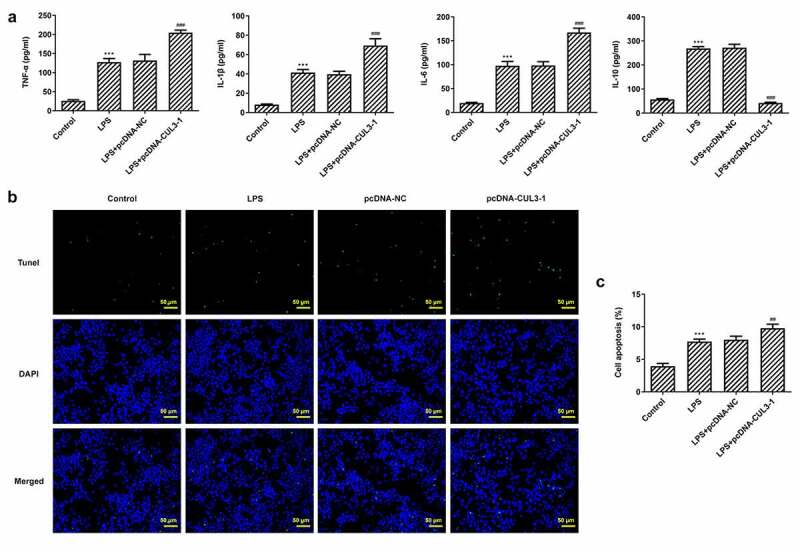
CUL3 overexpression promotes inflammation and apoptosis of PDLSCs treated with pg-LPS. (a) ELISA assay was performed to detect the effect of CUL3 overexpression on inflammatory factors (TNF-α, IL-6, IL-1β and IL-10) in PDLSCs treated with 1 μg/mL pg-LPS. (b) Tunel assay was performed to detected the effect of CUL3 overexpression on apoptosis of PDLSCs treated with 1 μg/mL pg-LPS. Magnification×200. (c) Statistical analysis of apoptosis. *p < 0.05, **p < 0.01 and ***p < 0.001 vs. pcDNA-NC. ***p < 0.001 vs Control, ##p < 0.01, ###p < 0.001 vs LPS+pcDNA-NC. CUL3: Cullin3.CUL3: Cullin3

**Figure 5.**
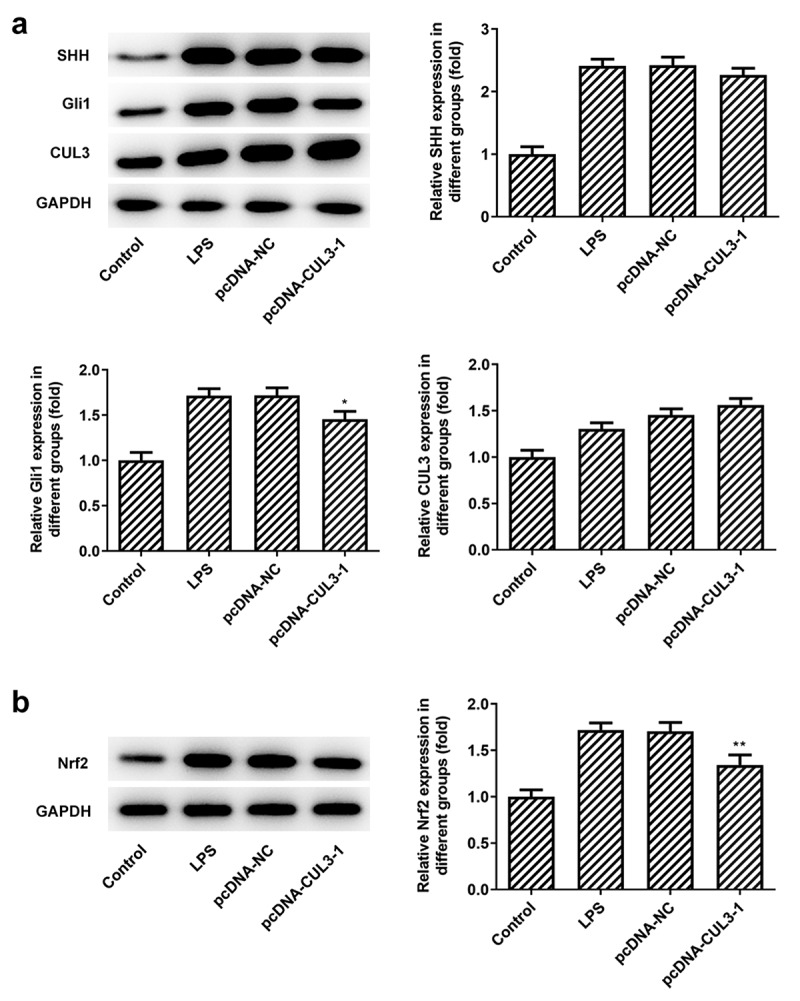
Effects of CUL3 overexpression on SHH pathway-related proteins and Nrf2 in PDLSCs treated with pg-LPS. (a) Western blot analysis was used to detect the effect of CUL3 overexpression on SHH, Gli1 and CUL3 in PDLSCs treated with 1 μg/mL pg-LPS. (b) Western blot analysis was used to detect the effect of CUL3 overexpression on Nrf2 and NQO1 in PDLSCs treated with 1 μg/mL pg-LPS. *p < 0.05 and **p < 0.01 vs. pcDNA-NC. ***p < 0.001 vs Control, #p < 0.05, ##p < 0.01 vs LPS+pcDNA-NC. CUL3: Cullin3.CUL3: Cullin3. CUL3: Cullin3

The inflammatory response of PDLSCs following transfection of pcDNA 3.1-CUL3 was evaluated using ELISA kits. It was noticed that 1 μg/ml Pg-LPS triggered high levels of TNF-α, IL-6 and IL-1β, meanwhile, CUL3 intensified the inflammatory reaction. By contrast, the level of IL-10 was tremendously decreased after the transfection of pcDNA 3.1-CUL3 (Figure 4(a)). Moreover, TUNEL staining demonstrated that LPS-induced cell apoptosis was further promoted by CUL3 overexpression (Figures 4(b) and 5(c)). Additionally, it was found that SHH, Gli1, Nrf2 and NQO1 were upregulated when transfected with 1 μg/ml Pg-LPS, while CUL3 overexpression partially ameliorated these effects (Figures 5(a)), indicating that CUL3 may promote inflammatory response and cell apoptosis through downregulating the expression of SHH, Gli1 and Nrf2.

## Discussion

PDLSCs are capable of achieving self-renewal and differentiation. They play a major role in repairing damaged dental tissues, thus, PDLSCs are considered to be of great use to periodontal reconstruction and regeneration [40]. Studies have shown that Pg-LPS can induce the inflammatory response of human periodontal ligament stem cells and form a periodontal disease model [36,37]. In the current study, we found that cell viability of PDLSCs was significantly reduced when exposed to 1 μg/ml Pg-LPS. Furthermore, numerous pro-inflammatory cytokines including TNF-α, IL-6 and IL-1β were produced after the stimulation of Pg-LPS. SHH is reported to potentially serve as a new therapeutic target for periodontitis and periodontal regeneration [41], the expression of which along with that of Gli1 in PDLSCs was examined in this study and was found to have an obvious increase after Pg-LPS stimulation. We know that CUL3 plays a negative role in the regulation of the SHH signaling pathway through the degradation of activated Gli by ubiquitination [38]. But in the study, gradient concentration of Pg-LPS triggered increase of CUL3, accompanied by increasing SHH and Gli1. One possible reason is that after induced by pg-LPS, cells produce acute inflammation. At this time, SHH signaling pathway is activated to protect cells from inflammatory damage. Therefore, after Pg-LPS induction, CUL3 was increased, and SHH and Gli1 were also increased. In basal condition, Keap1 binds Nrf2 to its BTB domain, and connects CUL3 to its Kelch domain. Nrf2 is maintained at a low level through CUL3-mediated ubiquitin-dependent degradation [24]. NRF escapes from degradation in response to stimulus, so it is released, thus regulating the expression of downstream target genes [42]. Our results showed that the expression of Nrf2 was increased when stimulated by Pg-LPS, at the same time, we also found that the overexpression of CUL3 in PDLSCs could impair cell viability and inhibit the differentiation and mineralization of PDLSCs. Moreover, inflammatory response was exacerbated and apoptotic cells was increased after overexpressing CUL3 with the existence of Pg-LPS. Of note, the levels of SHH, Gli1 and Nrf2 in PDLSCs was decreased when transfected with pcDNA3.1-CUL3, which suggested that SHH/Gli1 and Nrf2 mediated by CUL3 might participate in the inflammatory response, thus affecting the differentiation and mineralization of PDLSCs.

Keap1 is also one of the substrates of CUL3 [43]. CUL3 binds to KEAP1 and degrades it by ubiquitination, while Keap1/Nrf2 is an important pathway and mechanism in the regulation of oxidative stress and metabolism [44,45]. Keap/Nrf2 is also one of the key mechanisms in the NO metabolic pathway, and plays an important role in vascular smooth muscle injury, endothelial dysfunction, and arteriosclerosis [46]. The deletion or abnormal expression of CUL3 can lead to the failure of Keap1 to degrade normally, so that Nrf2 cannot dissociate normally from Keap1 outside the cytoplasm. Therefore, Nrf2 enters the nucleus and activates Antioxidant reaction element (ARE), which leads to the imbalance of NO production and metabolism, and then causes related lesions [47]. We found that the expression of Nrf2 in cells after overexpression of CUL3 was not significantly decreased, possibly because CUL3 could degrade Gli1 and lead to the decrease of Nrf2, and also degrade Keap1 and cause the activation of Nrf2. The former was dominant here, so the overall expression of Nrf was slightly reduced.

The limitations of our article were that knocking down the expression of CUL3 can make the experimental results more perfect. However, due to the length of this paper, we will further verify our experimental results by knocking down CUL3 in the following experiments. In summary, CUL3 was found to be involved in the inflammatory response of PDLSCs via orchestrating the expression of SHH signaling and Nrf2. Our paper provides a solid theoretical basis for the study of the mechanism of periodontitis and targeted therapy.

## Research highlights

CUL3 in periodontitis inhibits osteogenic differentiation.

CUL3 in periodontitis promotes cell inflammation and apoptosis.

CUL3 ubiquitination degrades Gli1 in periodontitis, thus negatively regulating SHH pathway

CUL3 degrades Gli1 and then down-regulates Nrf2 in periodontitis.

CUL3 degrades Keap1 and then activates Nrf2 in periodontitis.
